# Supplementary material for: Ubiquitin-Activating Enzyme E1 (UBA1) as a Prognostic Biomarker and Therapeutic Target in Breast Cancer: Insights into Immune Infiltration and Functional Implications
Source: Int J Mol Sci. 2024 Nov 26;25(23):12696. doi: 10.3390/ijms252312696 (PMC11641640; doi:10.3390/ijms252312696)
Supplement: Supplementary file 1 [file ijms-25-12696-s001.zip › ijms-3258428-supplementary.pdf]

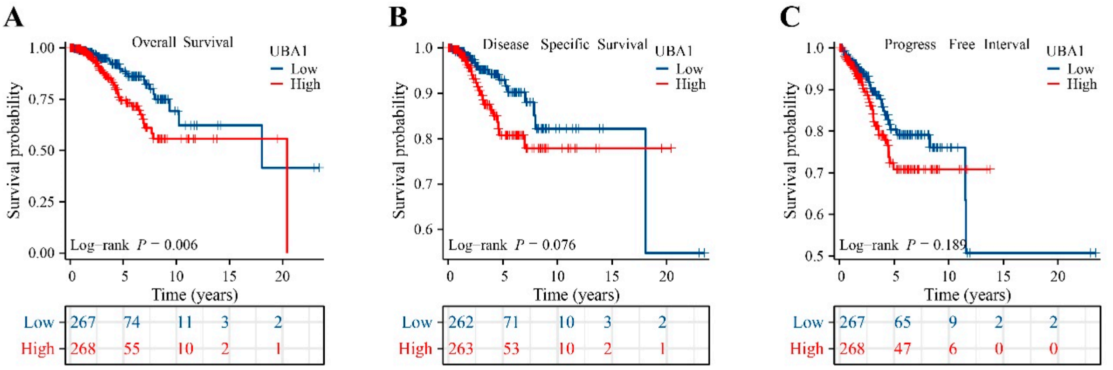

Figure S1. The log-rank test grouping by high (top 25%) and low (bottom 25%) expression UBA1 in BC patients.

Kaplan-Meier survival curve showing the prognostic values of UBA1 expression in individuals with BC (A) overall survival; (B) disease-specific survival; and (C) progress-free survival
